# Predictors of mortality and adverse events in patients with infective endocarditis: a retrospective real world study in a surgical centre

**DOI:** 10.1186/s12872-021-01853-6

**Published:** 2021-01-12

**Authors:** Valentina Scheggi, Irene Merilli, Rossella Marcucci, Stefano Del Pace, Iacopo Olivotto, Nicola Zoppetti, Nicole Ceschia, Valentina Andrei, Bruno Alterini, Pier Luigi Stefàno, Niccolò Marchionni

**Affiliations:** 1 Division of Cardiovascular and Perioperative Medicine, Cardiothoracovascular Department, Azienda Ospedaliero-Universitaria Careggi and University of Florence, Largo Brambilla 3, 50143 Florence, Italy; 2 Division of General Cardiology, Cardiothoracovascular Department, Azienda Ospedaliero-Universitaria Careggi and University of Florence, Florence, Italy; 3 Division of Cardiac Surgery, Cardiothoracovascular Department, Azienda Ospedaliero-Universitaria Careggi and University of Florence, Florence, Italy; 4 Institute of Applied Physics “Nello Carrara” (IFAC), National Research Council, Sesto Fiorentino, Italy

**Keywords:** Infective endocarditis, Prognostic factors, Outcome, Valvular dysfunction, Endocarditis, Mortality, Prognosis

## Abstract

**Purpose:**

Mortality in infective endocarditis (IE) is still high, and the long term prognosis remains uncertain. This study aimed to identify predictors of long-term mortality for any cause, adverse event rate, relapse rate, valvular and ventricular dysfunction at follow-up, in a real-world surgical centre.

**Methods:**

We retrospectively analyzed 363 consecutive episodes of IE (123 women, 34%) admitted to our department with a definite diagnosis of non-device-related IE. Median follow-up duration was 2.9 years. Primary endpoints were predictors of mortality, recurrent endocarditis, and major non-fatal adverse events (hospitalization for any cardiovascular cause, pace-maker implantation, new onset of atrial fibrillation, sternal dehiscence), and ventricular and valvular dysfunction at follow-up.

**Results:**

Multivariate analysis independent predictors of mortality showed age (HR per unit 1.031, *p* < 0.003), drug abuse (HR 3.5, *p* < 0.002), EUROSCORE II (HR per unit 1.017, *p* < 0.0006) and double valve infection (HR 2.3, *p* < 0.001) to be independent predictors of mortality, while streptococcal infection remained associated with a better prognosis (HR 0.5, *p* < 0.04). Major non-fatal adverse events were associated with age (HR 1.4, *p* < 0.022). New episodes of infection were correlated with S aureus infection (HR 4.8, *p* < 0.001), right-sided endocarditis (HR 7.4, *p* < 0.001), spondylodiscitis (HR 6.8, *p* < 0.004) and intravenous drug abuse (HR 10.3, *p* < 0.001). After multivariate analysis, only drug abuse was an independent predictor of new episodes of endocarditis (HR 8.5, *p* < 0.001). Echocardiographic follow-up, available in 95 cases, showed a worsening of left ventricular systolic function (*p* < 0.007); severe valvular dysfunction at follow-up was reported only in 4 patients, all of them had mitral IE (*p* < 0.03).

**Conclusions:**

The present study highlights some clinical, readily available factors that can be useful to stratify the prognosis of patients with IE.

## Background

Despite recent diagnostic and therapeutic advances, the mortality of IE remains high in most series, with in-hospital mortality even of 24% [1], and three-year mortality over 30% [2]. The literature reports some prognostic factors associated with higher mortality, such as advanced age, female gender [1–3], prosthetic valve endocarditis [4], Staphylococcus aureus aetiology [5], comorbidity [6–12], leucocytosis, hypoalbuminemia, C-reactive protein levels, elevated ERS [13], and IE complications [6].

Most published series are retrospective studies, each focusing on specific aspects. A multicenter cohort study evaluated predictors of hospital re-admissions in patients with infective endocarditis [14], one study included only patients with prosthetic valve IE [15], and another study included only surgically treated patients [16]. Still, there is a lack of consensus about which characteristics individuate patients with an adverse prognostic profile. The epidemiology of the disease has changed in recent decades. A progressive increase of the average patient age, a higher prevalence of cardiac devices and prostheses, and a rising incidence of nosocomial or procedure-related endocarditis have changed the microbiologic yield of the disease [17]. Therefore, an update on prognostic factors of infective endocarditis is necessary to consider the contemporary epidemiologic metamorphosis of this pathology. An accurate risk stratification could individuate patients who might benefit from a more aggressive strategy. This study aimed to investigate factors associated with a worse prognosis in a referral surgical centre.

## Methods

### Patient selection

**Fig. 1:**
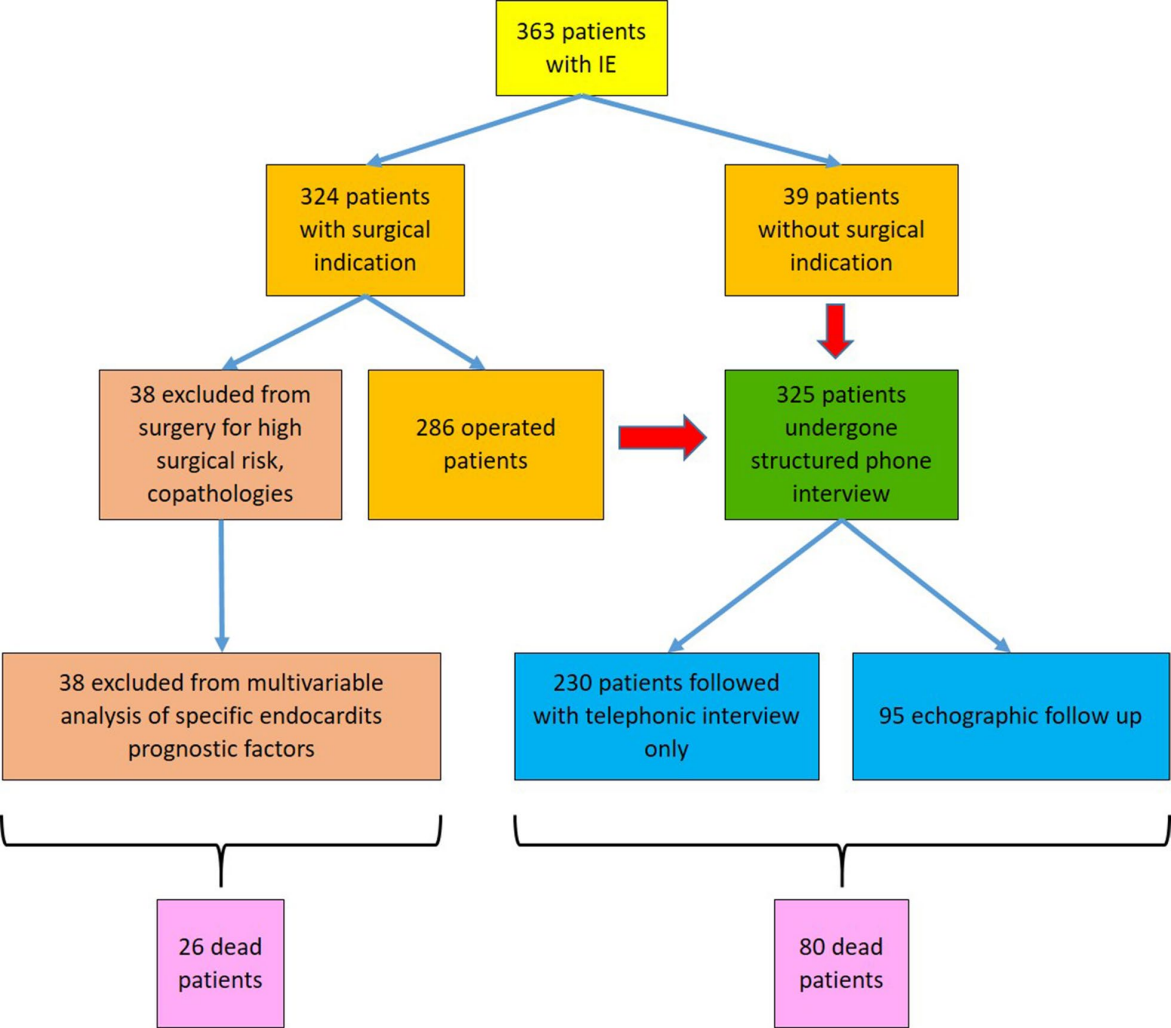
Flow chart of patient selection

We have created a local registry of patients affected by non-device-related IE, collecting retrospectively all the cases admitted to our department between January 2013 and December 2019 with a definite diagnosis of IE. Device-related IE was defined as an infection on cardiac devices, implants and grafts, other than valve prosthesis, documented by echocardiography. We excluded all patients with electro-catheter vegetation or cutaneous infection over the pocket area of the implanted device. The registry comprises 363 patients and 294 variables. We got data for analysis from electronic hospital charts and wholly anonymized them, as reported elsewhere [18]. The local Ethics Committee approved the study. We followed the current international IE guidelines for diagnostic work-up and treatment strategies [19]. Of the 363 patients with IE, 39 received only medical therapy because of the absence of surgical indication, 286 underwent surgical intervention associated with antibiotic treatment, and 38 were excluded from surgery despite surgical indication because of prohibitive general conditions. We excluded the latter group from the multivariate analysis of prognostic factors specific of IE since it identifies patients with extremely compromised conditions aside from IE, and their prognosis depended on critical basal conditions more than on IE itself (Fig. 1). Multivariate analysis of mortality was adjusted for the treatment received: medical therapy only, early surgery, or delayed surgery. We excluded procalcitonin and TAPSE from multivariate analysis, because of a high percentage of missing values (22 and 26% respectively). Variables included in the multivariate analysis were gender, age, diabetes, renal failure, history of drug abuse, the microbiologic agent involved, ejection fraction, the type of valve (native or prosthetic), double valve infection, the paravalvular extension of infection, severe valvular dysfunction, oral anticoagulant therapy, brain embolism at admission, EUROSCORE II and the presence of a pacemaker. We defined renal failure as GFR < 60 ml/min/1.73 m^2^.

### Follow-up

We calculated follow-up duration from the time of IE diagnosis. A structured phone interview updated the follow-up of all patients to July 2020. In a subset of 95 cases of the 325 patients included in the multivariate analysis, both echocardiographic and clinical evaluation were available (Fig. 1). Median follow-up duration was 2.9 years (SD 1.5), minimum 0.3, maximum 7 years.

### Study endpoints

Primary endpoints were predictors of mortality, recurrent endocarditis, major non-fatal adverse events (hospitalization for any cardiovascular cause, pace-maker implantation, new onset of atrial fibrillation, sternal dehiscence), worsening of left ventricular ejection fraction (LVEF), and valvular dysfunction during follow-up.

### Statistical analysis

We used the chi-square and the Mann–Whitney or Kruskal–Wallis tests to compare respectively proportions and continuous variables with normal or non-normal distributions. We performed univariate and multivariate analyses using logistic regression and general linear models. We used the Kaplan–Meier method to estimate the univariate cumulative incidence of events and event-free survival. All tests were 2-sided, and statistical significance was defined as a p-value < 0.05. We performed the analyses with SPSS 23.0 and R 3.6.3.

## Results

### Patient characteristics

We included 363 consecutive episodes of IE (123 women, 34%). Median follow-up was three years (0.3–7). We summarized the principal clinical and demographic characteristics of the 363 patients in Table 1. We reported in Table 2 the underlying valvular disease and the degree of valvular dysfunction.

**Table 1:** Demographic, clinical and echocardiographic characteristics of 363 patients with IE

*Demographic and clinical characteristics of 363 patients with IE*	
Age (years), mean ± SD	65 ± 15
Gender (women), n (%)	123 (34)
BMI, mean ± SD	25.0 ± 4.1
Renal failure, n (%)	96 (26)
Mild n (%)	41 (43)
Moderate n (%)	34 (35)
Severe n (%)	14 (15)
On dialysis n (%)	7 (7)
Arterial hypertension, n (%)	212 (59)
Previous malignancies, n (%)	79 (22)
Drug abuse, n (%)	43 (12)
Diabetes, n (%)	68 (18)
Dyslipidemia, n (%)	109 (30)
Pacemaker, n (%)	41 (11)
Oral anticoagulant therapy, n (%)	91 (25)
*Clinical and echocardiographic characteristics of 363 episodes of IE*	
Prosthetic valve, n (%)	132 (36)
First episode of IE, n (%)	330 (90)
Systemic embolism, n (%)	141 (39)
Cerebral embolism, n (%)	67 (47)
Perivalvular extension, n (%)	84 (23)
Severe valvular dysfunction, n (%)	172 (47)
Vegetation length (mm), mean ± SD	9.1 ± 7.6
Ejection Fraction (%), mean ± SD	56.7 ± 10.0
TAPSE (mm), mean ± SD	20.8 ± 5.6
Euroscore II	12.4 ± 16.5

**Table 2:** Underlying valvular disease in 363 episodes of IE

Type of valve n (%)	Site of infection n (%)	Degree of valvular dysfunction (stenosis/regurgitation)		
		Absent n (%)	Mild or moderate n (%)	Severe n (%)
Native 231 (63%)	Aortic 106 (29%)	17 (5)	30 (8)	59 (16)
	Mitral 108 (29%)	10 (3)	34 (9)	64 (17)
	Tricuspid 17 (5%)	2 (1)	6 (2)	9 (2)
Prosthetic 132 (37%)	Aortic 87 (24%)	23 (6)	38 (11)	26 (7
	Mitral 39 (11%)	8 (2)	19 (5)	12 (3)
	Tricuspid 6 (2%)	0 (0)	4 (2)	2 (1)

### Microbiology

Blood cultures were positive in 80% of cases. We summarized the microbiologic yield in Table 3. A presumable bacterial access location was present in 241 (66%) patients. The access location was cutaneous in 71 patients (29%), nosocomial in 57 (24%), gastroenteric in 51 (21%), urinary in 34 (14%), and oropharyngeal in 28 (12%). Since the prevalence of streptococcal infection was 16%, oropharyngeal access was probably underdiagnosed.

**Table 3:** Microbiological yield of blood cultures in 363 episodes of IE

Etiologic agent	N (%)
Streptococcus Viridans	62 (17)
Streptococcus Bovis	25 (7)
Staphylococcus Aureus	64 (18)
Negative Coagulase Staphylococci	46 (13)
Enterococci	65 (18)
Other	27 (7)
Negative cultures	74 (20)
Total	363 (100)

### Surgical treatment

A total of 286 (79%) episodes of IE underwent surgical intervention; 246 (86%) underwent valve replacement and 40 valve repair (14%). Seventy-seven (21%) patients were treated conservatively, 39 due to the absence of surgical indication, and 38 due to prohibitive surgical risk. Surgery was performed within 14 days from diagnosis in 222 (78%) patients and later in the remaining 64 (22%); in these patients, surgery was delayed because of neurologic complications or a delayed referral from peripheral hospitals.

The purpose of this study was to evaluate prognostic factors at baseline, rather than studying a specific treatment or therapeutic strategy. We didn’t deepen comparison among medical therapy, early surgery and delayed surgery. On the other hand, exclusion from surgery in patients with surgical indication had disproportionately high mortality: in this subgroup, mortality at 30 days extrapolated from Kaplan–Meier curves was 33.8% (95% CI 17–47.1), and one-year mortality was 64.9% (95% CI 46.1–77.2). Indeed, these patients had prohibitive general conditions, aside from IE. As stated in the method section, we, therefore, excluded this subgroup of patients from the multivariate analysis of mortality.

Surgical intervention was performed less frequently in women (*p* < 0.027), elderly (*p* < 0.037) and in patients with a low ejection fraction (*p* < 0.001); at multivariate analysis female gender was an independent factor for exclusion from surgery for absence of surgical indications (OR 0.44, 95% CI 0.21–0.91, *p* < 0.028), while ejection fraction (OR per increasing unit 0.94, 95% CI 0.91–0.97, *p* < 0.001) and age (OR per increasing unit 1.05, 95% CI 1.02–1.08, *p* < 0.001) were independent predictors for exclusion from surgery when indicated, with an inverse and direct relation respectively.

### Mortality

**Fig. 2:**
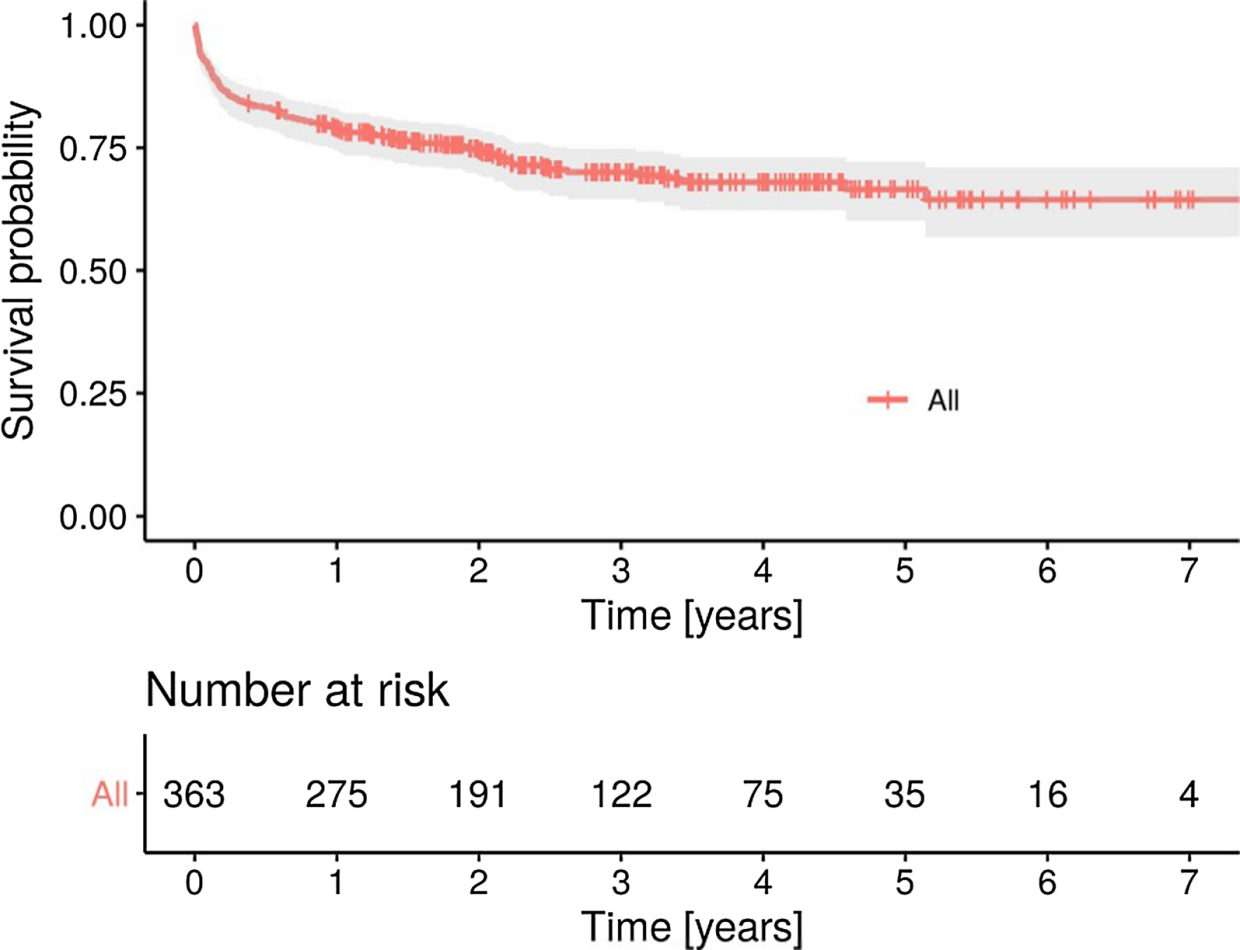
Kaplan–Meier analysis of survival probability of 363 patients with infective endocarditis

**Fig. 3:**
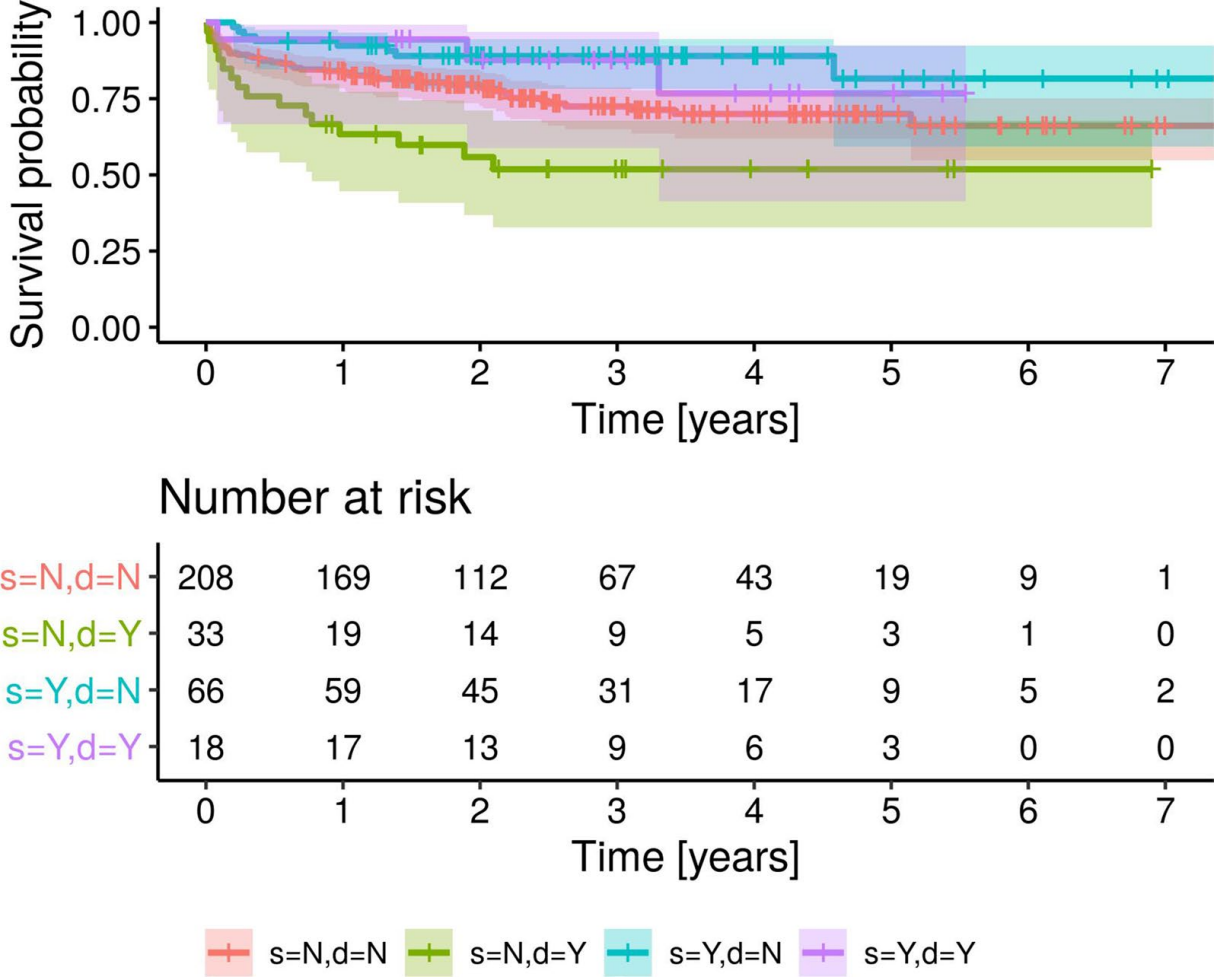
Multivariate Kaplan–Meier analysis of survival probability of 325 patients with infective endocarditis with (Y) or without (N) double valve infection (d) and with (Y) or without (N) Streptococcal infection (s)

After extrapolation from Kaplan–Meier curves, overall all-cause thirty-day mortality was 7.7% (95% CI 4.9–10.4), and all-cause three-year mortality was 29.9% (95% CI 24.7–34.8) (Fig. 2). We reported the predictors of death for any cause at univariate analysis in Table 4. The independent predictors of mortality were age, drug abuse, EUROSCORE II and double valve infection, while streptococcal infection confirmed to be associated with a better prognosis (Table 4). Given a type I error rate of 0.05, the detected differences had a power level > 0.8, except for the association of streptococcal infection with lower mortality, that was characterized by a power of 0.5. Figure 3 shows the multivariate survival curves of double valve infection and streptococcal infection.

**Table 4:** Univariate and multivariate analysis of predictors of mortality for any cause

	HR	95% CI		*p*
*Univariate analysis (n* *=* *363)*				
Female gender	1.2	1.0	1.4	0.023
Age > 70 years	2.1	1.4	3.2	0.001
Euroscore II > 10	3.1	2.1	4.6	0.001
Chronic renal failure	1.9	1.3	2.9	0.001
Diabetes	1.7	1.1	2.7	0.009
Oral anticoagulant therapy	1.7	1.2	2.7	0.005
Presence of pacemaker	2.2	1.4	3.6	0.001
Ejection fraction < 40%	3.1	1.8	5.4	0.001
TAPSE < 19	2.4	1.5	3.9	0.001
Double valve infection	1.8	1.2	2.9	0.007
Prosthetic infection	1.7	1.1	2.5	0.015
Procalcitonin levels > 0.22 mg/dl	1.9	1.2	3.0	0.003
PCR > 83 mg/dl	1.6	1.0	2.5	0.037
Enterococcal infection	1.6	1.0	2.5	0.022
Streptococcal infection	0.34	0.1	0.6	0.002
Exclusion from surgery if indicated	7.4	3.2	17.3	0.001
*Multivariate analysis (n* *=* *325)*				
Age*	1.033	1.01	1.056	0.003
Drug abuse	3.40	1.41	8.13	0.006
EUROSCORE II*	1.017	1.009	1.025	0.0006
Double valve infection	2.07	1.21	3.54	0.008
Streptococcal infection	0.49	0.26	0.92	0.028

^*^HR per unit

### Major non-fatal adverse event rate and new infection rate

During follow-up, 112 (34%) patients had had one or more major non-fatal adverse events. Age, hypertension, and history of cancer predicted major non-fatal adverse events at three years. At multivariate analysis, only age was independently associated with major non-fatal adverse events (Table 5). Nineteen patients (6%) had one or more recurrent episodes of IE. S aureus infection, right-sided endocarditis, spondylodiscitis, and drug abuse predicted recurrent infection. After multivariate analysis, only drug abuse was an independent predictor of new episodes of endocarditis (Table 5).

**Table 5:** Univariate and multivariate analysis of predictors of major non-fatal adverse events and episodes of recurrent infective endocarditis

	HR	95% CI		*p*
*Major non-fatal adverse events (112 patients), univariate analysis*				
Age	1.02	1.00	1.03	0.001
Hypertension	1.46	1.06	2.03	0.02
History of cancer	1.56	1.11	2.20	0.009
*Major non-fatal adverse events (112 patients), multivariate analysis*				
Age	1.49	1.06	2.118	0.022
*Recurrent episodes of IE (19 patients), univariate analysis*				
Staphylococcus aureus infection	4.8	1.9	11.9	0.001
Right-sided endocarditis	7.4	2.9	18.9	0.001
Spondylodiscitis	6.8	2.6	17.8	0.004
Drug abuse	10.3	4.1	25.6	0.001
*Recurrent episodes of IE (19 patients), multivariate analysis*				
Drug abuse	8.5	2.3	31	0.001

### Echocardiographic follow-up

We performed echocardiographic follow-up in a subset of 95 patients. We used the PISA method for quantitative assessment of valve regurgitation and the Simpson method from the apical 4-chamber view for the quantitative evaluation of Left ventricular ejection fraction (LVEF). We reported clinical and echocardiographic data of this subset of patients in Table 6. The subset of patients undergone echographic follow-up is not clinically representative of the whole cohort, since patients willing to come to the visit were younger and had better general conditions (synthetically expressed by EUROSCORE II at diagnosis). On the other hand, echocardiographic parameters were not significantly different between the two groups (Table 7), and results are extensible to the entire cohort. The average EF at baseline was 59.8 (SD 8.13) while the follow-up EF was 57.46 (SD 8.88); the EF distributions are not normal (Shapiro test *p* = 0.036 and *p* = 0.003). These differences are not significant considering baseline and follow-up measures as independent (Mann–Whitney test, *p* < 0.093), while they are significant when considering repeated measures (Wilcoxon signed-rank test, *p* < 0.007). Predictors of worsening EF will be the object of investigation in a thorough study dedicated to echocardiographic follow-up. Only four patients reported severe valvular dysfunction at follow-up, all of them had mitral IE (*p* < 0.03), one treated conservatively because of refusal of surgery, one undergone valve repair, and two valve replacement.

**Table 6:** Clinical and echocardiographic data of patients undergone to echocardiographic follow-up (n = 95)

	Site of infection			*p*
	Aortic (n = 50)	Mitral (n = 39)	Tricuspid (n = 6)	
Gender (women), n (%)	9 (18)	7 (18)	0 (0)	0.5
Age (years), mean ± SD	61.5 ± 14.8	60.3 ± 12.2	61.6 ± 12.5	0.7
Severe valvular dysfunction, n (%)	23 (46)	25 (64)	3 (50)	0.2
Paravalvular extension, n (%)	13 (26)	5 (13)	0 (0)	0.1
Vegetation length (mm), mean ± SD	7.7 ± 6.2	10.2 ± 7.9	26.3 ± 13.3	0.008
Second infected valve, n (%)	4 (8)	5 (13)	1 (17)	0.6
Ejection Fraction (%), mean ± SD	59.1 ± 8.0	60.9 ± 8.5	58.3 ± 6.6	0.5
TAPSE (mm), mean ± SD	20.8 ± 6.2	23.2 ± 5.3	21.2 ± 6.9	0.2
Euroscore II	7.3 ± 6.3	5.6 ± 9.1	4.3 ± 2.5	0.035
Surgery, n (%)	42 (84)	34 (87)	6 (100)	0.5
Ejection Fraction (%) at follow-up, mean ± SD	58.9 ± 8.3	55.9 ± 9.1	55.1 ± 11.0	0.2
Severe valvular dysfunction at follow-up, n (%)	0 (0)	4 (10)	0 (0)	0.03

**Table 7:** Comparison between patients undergone or not to echocardiographic follow-up

Echocardiographic follow-up	No (n = 268)	Yes (n = 95)	*p* value
Female gender, n (%)	107 (40)	16 (17)	< 0.001
Age (years), mean ± SD	66.8 ± 15.1	61.0 ± 13.5	< 0.001
Euroscore II, mean ± SD	14.5 ± 18.2	6.4 ± 7.5	< 0.001
Site of infection			NS
Aortic	143 (53)	50 (53)	NS
Mitral	108 (40)	41 (41)	NS
Tricuspidal	17 (7)	6 (6)	NS
Ejection Fraction (%), mean ± SD	55.6 ± 10.3	59.8 ± 8.1	NS
TAPSE (mm), mean ± SD	20.3 ± 5.4	21.9 ± 5.9	NS
Vegetation length (mm), mean ± SD	8.8 ± 7.2	9.9 ± 8.6	NS

## Discussion

The three-year mortality rate of IE in the literature often exceeds 30% [2]. The overall mortality rate at three years in our series was 29%. We found several, commonly available predictors of higher mortality in endocarditis, that can be useful for clinicians to stratify patient prognosis better. The principal adverse prognostic factors belong to three categories: clinical features, echocardiographic parameters, and microbiologic aetiology.

### Clinical features

Clinical characteristics associated with an adverse prognosis were female gender, age > 70 years, EUROSCORE II, chronic renal failure, diabetes, and drug abuse. Gender differences are attributable to a variety of factors, including comorbidities or inherent physiologic differences [1]. IE occurs in males more frequently, with a 2:1 to 9:1 ratio. Estrogens may play a protective role against endothelial damage. Moreover, females tend to encounter heart disease at an older age and have a higher incidence of comorbid conditions, which may result in a worse outcome [2, 3]. Deepening the analysis, we found that the female gender was not an independent predictor of adverse prognosis, as opposed to age and EUROSCORE II, that confirmed to be independent predictors at multivariate analysis. Despite the availability of recent IE-specific scores and considering the trade-off between the indexes, the logistic EUROSCORE II seemed to be the best predictor of mortality risk in the literature [5–8]. Relatively to age, the epidemiology of IE patients has changed. Currently, most of IE patients are elderly, frail, and with multiple comorbidities [9–12]. Drug addiction is also an independent predictor of adverse prognosis in IE and the major risk factor for recurring IE, consistently with other studies [13–21].

### Echocardiographic parameters

Echocardiographic predictors of a worse prognosis were double valve infection, ejection fraction < 40%, and prosthetic infection. The value of echocardiographic findings in predicting outcome in infective endocarditis is emergent [22]. Heart failure is associated with a worse outcome in IE [15, 23], as well as echocardiographic parameters showing right or left ventricular dysfunction [24–26]. Prosthetic infection is a known adverse prognostic factor, both for biological and mechanical valves [16, 19]. Double valve endocarditis is a rare condition that is independently associated with an adverse outcome. This association was previously described in surgical series [27, 28], while we found this is a marker of worse outcome independently of surgery. The mechanisms of the spread of the infection differ whether endocarditis is only left-sided or bilateral (also involving tricuspid valve). Left-sided bivalvular endocarditis is often due to a secondary mitral lesion following primary aortic endocarditis. Multivalvular endocarditis is frequently responsible for severe heart failure. Coherently with our data, the study of Sulton-Suty proofs the importance of early diagnosis of endocarditis to avoid the spread of the infection to more than one valve [29]. The involvement of multiple valves leads to a higher rate of complications and thus, mortality. Moreover, biventricular IE is an uncommon condition with no specific guidelines for treatment [30].

Echocardiographic follow-up showed a mild but significant average worsening of left ventricular systolic function; we will address predictors of lowering of LVEF in an incoming study.

### Microbiologic aetiology

Finally, microbiologic aetiology can identify patients with different prognosis. Enterococcal infection is associated with a worse outcome, while streptococcal IE seems to be protective. Chirouze’s study [31] found that enterococcal IE is healthcare-associated in 25% of cases and involves prosthetic valves in 30%. In our series, the enterococcal infection had a worse prognosis but was not an independent predictor of mortality at multivariate analysis, reflecting the specific epidemiology of this germ. On the other hand, Streptococcal infection was independently associated with a better prognosis, suggesting a less aggressive infection of this pathogen. These data emphasize the prognostic relevance of microorganism characterization in IE.

Our study has some limitations: first, changes in the clinical management of IE may have occurred during the long study period. Second, it is a real-world single-centre experience. Finally, our study has a potential referral bias, since we have conducted it in a high-volume surgical centre; therefore, the percentage of patients with a surgical indication in disproportionately high.

## Conclusions

In our series, the independent predictors of mortality were age, drug abuse, EUROSCORE II, and double valve infection, while the streptococcal infection had a better prognosis. Age predicted major non-fatal adverse events. Drug abuse was the only independent predictor of new episodes of endocarditis. Our results demonstrate some readily available parameters that identify IE patients at an increased risk of adverse prognosis. Accurate stratification of the prognosis could orient towards more aggressive management in selected patients.

## Data Availability

The datasets used and/or analyzed during the current study are available from the corresponding author on reasonable request.
